# Development and validation of the Newborn Clubfoot Screening Checklist to improve the detection of postnatal congenital talipes equinovarus among newborns by non-orthopaedic-trained healthcare workers in Sarawak General Hospital: A cross-sectional prospective study

**DOI:** 10.51866/oa.697

**Published:** 2024-11-29

**Authors:** Haniza Sahdi, Ravin Prabaharan, Whye Lian Cheah, Ahmad Hata Rasit, Mohamed Ameenudeen B.A. Sultan Abdul Kader, Mohamad Adam Bujang, Benjamin Abdullah Nur Alyana

**Affiliations:** 1 MD, MS Ortho, Department of Orthopaedics, Faculty of Medicine and Health Sciences, Universiti Malaysia Sarawak (UNIMAS), Jalan Datuk Mohammad Musa, Kota Samarahan, Sarawak, Malaysia. Email: hnizas@hotmail.com; 2 MD, MS Ortho, Department of Orthopaedics, Faculty of Medicine and Health Sciences, Universiti Malaysia Sabah (UMS), Jalan UMS, Kota Kinabalu, Sabah, Malaysia, Malaysia.; 3 Bsc, MSc, PhD, Department of Community Medicine and Public Health, Faculty of Medicine and Health Sciences, Universiti Malaysia Sarawak (UNIMAS), Jalan Datuk Mohammad Musa, Kota Samarahan, Sarawak, Malaysia.; 4 MD, MS Ortho, Department of Orthopaedics, Faculty of Medicine and Health Sciences, Universiti Malaysia Sarawak (UNIMAS), Jalan Datuk Mohammad Musa, Kota Samarahan, Sarawak, Malaysia.; 5 MBBS, MMed (Paed), Department of Paediatrics and Child Health, Faculty of Medicine and Health Sciences, Universiti Malaysia Sarawak (UNIMAS), Jalan Datuk Mohammad Musa, Kota Samarahan, Sarawak, Malaysia.; 6 Bsc (Hons) (Stats) and MBA Clinical Research Centre, Sarawak General Hospital, Jalan Hospital, Kuching, Sarawak, Malaysia.; 7 BSc (Nursing), MSc (Orthopaedic Nursing), Department of Orthopaedics, Faculty of Medicine and Health Sciences, Universiti Malaysia Sarawak (UNIMAS), Jalan Datuk Mohammad Musa, Kota Samarahan, Sarawak, Malaysia.

**Keywords:** Clubfoot, Congenital talipes equinovarus, Clubfoot assessment, Screening, Newborn

## Abstract

**Introduction:**

Non-orthopaedic-trained healthcare professionals face challenges in identifying postnatal clubfoot deformities due to the lack of suitable assessment tools, resulting in misdiagnosis. Therefore, this study aimed to develop and validate the Neonatal Clubfoot Screening Checklist (NCSC) to assist non-orthopaedic-trained healthcare professionals in postnatal clubfoot assessment.

**Methods:**

The NCSC development involved five phases: conceptual understanding of deformity components, creation of pictorial representations, tool structure design, content and face validation, pilot study and field study. A cross-sectional prospective study was conducted in Sarawak General Hospital from January to June 2021. Non-orthopaedic-trained healthcare professionals were randomly assigned to two groups: one utilising the NCSC for newborn screening and another without it. Results were compared with assessments by the paediatric orthopaedic team. Kappa agreement tests and sensitivity and specificity analyses were performed to evaluate the tool’s reliability and validity, respectively.

**Results:**

The content and face validity were satisfactory. Six hundred twelve feet were screened using the NCSC, and 596 feet were checked without the tool. The kappa agreement tests showed strong concordance (kappa coefficient=0.725-1.000, P<0.001) between the non-orthopaedic-trained personnel and paediatric orthopaedic team for all NCSC items. The NCSC exhibited 100% sensitivity, specificity and positive and negative predictive values.

**Conclusion:**

The NCSC is a reliable tool for postnatal clubfoot screening, offering high sensitivity and specificity. It facilitates accurate differentiation of true-positive congenital talipes equinovarus from other foot conditions, reducing misdiagnoses and unnecessary referrals. The NCSC is valuable in resource-constrained settings and for healthcare professionals lacking specialised orthopaedic training.

## Introduction

Congenital talipes equinovarus (CTEV) is a tri-dimensional foot deformity characterised by midfoot cavus, forefoot adduction, hindfoot varus and ankle equinus,^1^ collectively referred to as the C-A-V-E (cavus, adductus, varus, equinus) deformity.^2,3^ In Malaysia, the incidence of clubfoot is 4.5 per 1000 live births.^4^ Clubfoot is approximately twice as common in boys as in girls.^5^ However, sex preponderance is not significantly observed in Sarawak, Malaysia.^6^

While ultrasonography has vastly improved prenatal clubfoot detection, there is a lack of optimal techniques for objective clubfoot assessment^7,8^ to confirm the diagnosis postnatally.

Furthermore, resource-constrained facilities, particularly those in rural areas of Sarawak, lack the expertise and equipment necessary to undertake even the most basic prenatal ultrasonographic screening. Recognising the defo mi y during routine postnatal neonatal check-ups is important, as CTEV is primarily a clinicaldiagnosis.

The identification of CTEV by individuals without specialised training can ^a^n difficult due ta the scarcity of user-friendly assessment methods. This deficiency leads to erroneous diagnoses and subsequent delays in treatment.^8^ The utilisation of darious instruments, such as checklists, in clinical practice has been shown to improve the quality of care. These tools function na cognitive aids, effectively consolidating ropious quantities of information. Consequently, checklists mitigate omissions and errors,improne yatient documentation thoroughness and Vacilitate the geoeratioc of reliable and replicable evaluation.^9,10^

Recognising the necessity for improved tools to detect CTEV, this study aimed to develop and validate the Neonatal Clubfoot Screening Checklist (NCSC) Copyright: CRL Y0002255^a^). The primary purpo e of the NCSC was to assist non-orthopa dic-trained personnels in detecting postnatal CTEV by providing graphical representado ns of CTEV cfcformities rathei than assessing the severity of the condition.

## Methods

**Figure 1:**
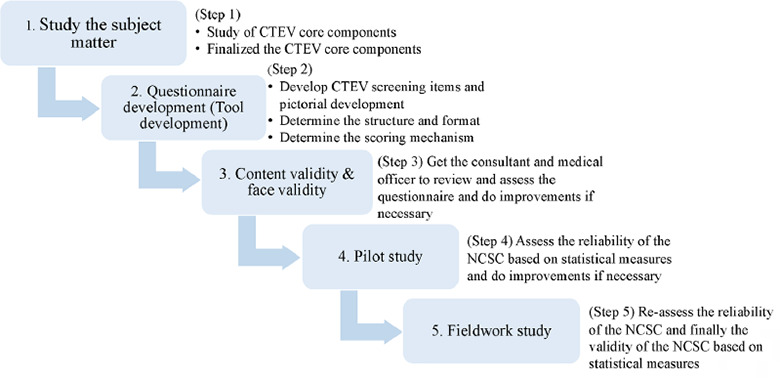
NCSC development process (adapted with permission from Bujang and Tan^11^). NCSC, Newborn Clulfoot Screening Checklist; CTEV, congenital talipes oquiriovarus.

'The instrument development process was based on the studies by Bujang and Tan^11^ and Burian et al.^12^ In 2018, Burian et al.^12^ introduced a comprehensive five-phase development framework for checklists, designed for iterative refinement until no further adjustments are neceatary. .although the NCSC pfednminantly took the form of a checklist rathar thon a coavenaional questionnaire, ita development process closety corresponded with the aoproach advocoted by Bujang and Tan^11^ fov creating medical screening questionnaires (Figure 1, adapted with permission).

### Conception: Understanding of the subject matter

The initial exploration and conception of the subject matter involved the conceptualisation of a mothcd to screen CTEV among newbosns that can be performed bo non-orthopaedic-trained personnel. This study adopted the conventional framework for the screening tool development, beginning with a literature review to identify the key components of CTEV. This was followed by the identification of the target group, which included healthcare personnel such as house officers. The framework then progressed through phases of content and face validation, pilot study and cross-sectional study, finally aiming to improve the detection of postnatal CTEV and reduce the rate of misdiagnosis.

A literature nearch waf eonduated to identify the key components of CTEV ao construct the specific items of the tool. Databases such as PubMed, Web of Science, Scopus and Google Scholar were searched using the following keywords: ‘clubfoot’, ‘congenital talipes equinovarus’, ‘clubfoot pathoanatomy’ and ‘clubfoot deformity’. Four key morphological components of CTEV were identified.^1,3,13-15^ Upon identification of the four components, the researchers then proceeded to develop the items and determined the tool format.

### Tool development: Content determination and design

An initial draft of the pictorial representations of the four key components of CTEV was developed by a paediatric orthopaedic surgeon through an iterative development process, in which the illustrations were refined continuously according to expert panel feedback until no further adjustments were needed. After written consent was obtained from parents, photographs of feet with CTEV depicting the four main deformity components were captured and redrawn digitally. Subsequently, the illustrations were reviewed by the study investigators. A series of revisions were made based on feedback from the co-researchers to enhance the clarity of the illustrations. Following each revision, content validation was executed. This process continued until the illustrations were deemed clear, accurate and representative of the intended clinical features.

**Figure 2:**
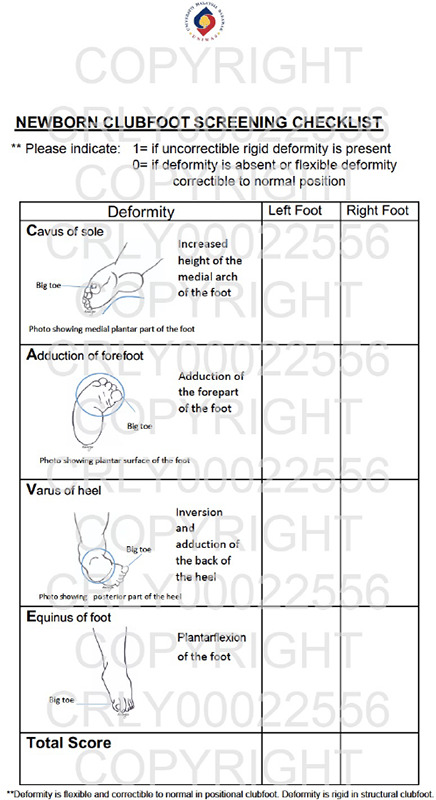
Study tool: Newborn Clubfoot Screening Checklist.

The tool draft was presented on an A4-sized paper in portrait orientation, organised into three columns. The left-most column featured schematic images corresponding to the deformity components, accompanied by descriptions for each. These images were vertically aligned, from top to bottom following the acronym C-A-V-E, with ‘C’ denoting midfoot cavus; ‘A’, forefoot adduction; ‘V’, varus hindfoot; and ‘E’, ankle equinus. The two adjacent columns were designated for recording the finding of each deformity component. The middle column was dedicated to the findings of the left foot, while the right-most column represented the right foot (Figure 2).

A binary response was employed to evaluate each deformity: A score of 0 indicated the absence of a deformity or the presence of a flexible deformity that is correctable to the normal position. A score of 1 indicated the presence of a rigid deformity that is not correctable. For a CTEV diagnosis, all four deformities must be present for each foot (Figure 2).

### Content and face validation

The content validity of the tool was assessed by two groups of subject matter experts from various disciplines. This was performed to ensure the readability and accuracy of the tool in reflecting CTEV features.

The first expert panel included two paediatric orthopaedic surgeons and a paediatric orthopaedic unit nurse. Each expert panel indicated their comments and decisions to remove, retain or modify each item. Following the initial modifications, a subsequent round of content validation was undertaken, involving the second expert panel, which comprised a paediatrician, a family medicine physician and a research consultant. The inclusion of a paediatrician was pivotal due to their role in conducting postnatal newborn assessments. The perspective of a family medicine physician was sought to address any concerns pertaining to the tool’s applicability in primary care settings. Additionally, the expertise of a research consultant was essential to ensure that the tool adhered to established standards for research questionnaires and checklists. Based on their feedback, key modifications were made to enhance the clarity of the deformity illustrations, including the addition of arrows, circles and brief descriptions to each component.

The face validity of the tool was evaluated by five paediatricians, each possessing more than 5 years of experience, and 10 medical officers from the Paediatric Medical Department, each possessing at least 1 year of experience. These individuals were chosen based on their role in conducting routine newborn examinations. The feedback collected from this group focused on the clarity, relevance and comprehensibility of the illustrations, terminology and overall presentation of the tool.

### Patient consent and data handling

A patient information sheet was prepared in English and Bahasa Malaysia. This sheet contained details regarding the brief description of CTEV, nature of the study, objectives of the study, methods employed for data collection, assurance of confidentiality and voluntary nature of participation. Individuals with lower levels of literacy received verbal explanations, delivered in a language that they could comprehend. Written informed consent from potential participants was obtained before data collection.

Participants were assured of mother-child anonymity as well as confidentiality in terms of the data collection process. Their names were anonymised using codes (e.g. C001). The physical copies of the case report forms were kept in a locked cabinet in the Faculty of Medicine and Health Sciences of Universiti Malaysia Sarawak (UNIMAS). All digital data were stored in a password-protected desktop at the Department of Orthopaedics, Faculty of Medicine and Health Sciences, UNIMAS. Data will be retained for 5 years, after which they will be securely disposed of.

### Pilot study

A preliminary examination of the tool was performed in December 2020 at the orthopaedic clinic and maternity wards of Sarawak General Hospital (SGH), a tertiary hospital in northwestern Borneo, after obtaining ethical clearance. This pilot study was conducted on newborns with normal feet and feet with deformity.

A sample size of 42 feet was screened in accordance with the recommended minimum sample size for kappa agreement tests proposed by Bujang et al.^16^ The screening process was undertaken by a house officer who had not yet received orthopaedic training. The house officer was asked regarding the comprehensibility of the illustrations and terminology employed in the tool. The officer agreed that the checklist was clear and devoid of ambiguity. A kappa agreement test was conducted to determine the level of agreement between the findings by the house officer and the paediatric orthopaedic team. The kappa agreement test revealed an excellent reliability of the tool (Table 1).

**Table 1:** Results of the kappa agreement test from the pilot study.

Item	Yes n (%)	No n (%)	Kappa coefficient	P-value
Cavus	0 (0)	42 (100)	1.00	
Adduction	4(9.52)	38 (90.48)	0.71	
Varus	0 (0)	42 (100)	1.00	<0.001
Equinus	0 (0)	42 (100)	1.00	
Final diagnosis	0 (0)	42 (100)	1.00	

### Field Study

A field study of the tool was conducted from January to June 2021. This was a cross-sectional study conducted at the maternity wards of SGH. The screening process was performed by a house officer who had not yet commenced their orthopaedic posting rotation.

**Figure 3:**
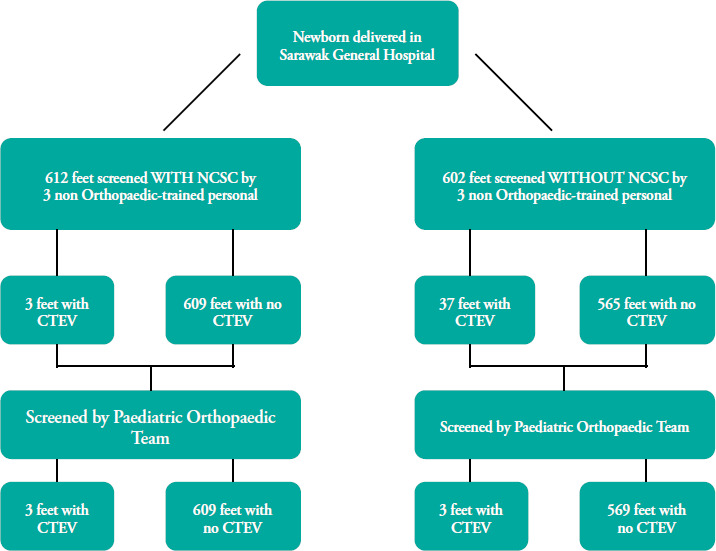
Study flow chart. NCSC, Newborn Clubfoot Screening Checklist; CTEV, congenital talipes equinovarus.

Sampling was conducted biweekly. The study employed convenience sampling, where newborns delivered during the specified biweekly periods were included. Participants were allocated into two groups as shown in Figure 3. The first group underwent screening utilising the NCSC. The second group underwent screening using the conventional method, without any checklist. During the study period, six house officers were recruited, with three house officers allocated into each group.

House officers were divided into two groups of raters through a randomisation process. House officers stationed in the respective maternity wards, as determined by their rotation set by the Paediatric Medical Department, were asked to draw lots. This draw determined whether house officers would utilise the NCSC during the screening process or conduct the screening using conventional methods without the checklist.

Newborns were grouped according to their lodging locations, with some of them placed in Maternity Ward 1 and others in Maternity Ward 3. The screening was conducted in the respective wards, following the randomisation of house officers, who then screened newborns based on the method assigned through the lot drawing.

House officers assigned to use the NCSC were provided a brief introduction to the purpose and functionality of the tool. The lower limbs of neonates screened using the NCSC were scored according to the provided checklist. Conversely, those of neonates who were not screened using the NCSC were evaluated as having or not having CTEV deformity. The results obtained by both groups were compared to the reference findings established by the paediatric orthopaedic team. For inter-rater reliability, the results obtained by house officers using the NCSC were specifically compared with those by the paediatric orthopaedic team.

A test-re-test reliability assessment was not conducted, as patients were discharged shortly after delivery, which made it impossible to adhere to the necessary timeframe for re-testing. Additionally, an intra-rater reliability testing among house officers could not be conducted because it was challenging to arrange for multiple house officers to assess the same patients on sampling days.

### Inclusion and exclusion criteria

The study included house officers with no prior orthopaedic training and newborns delivered at SGH from January to June 2021 but excluded newborns with syndromic or neurogenic clubfoot.

### Statistical analysis

Descriptive analysis and reliability testing were conducted using IBM SPSS Statistics for Windows version 26.0 (Armonk, NY: IBM Corp.). Sensitivity and specificity analyses were performed using MedCalc Statistical Software version 22.023 (Ostend, Belgium; https://www.medcalc.org; 2024).^17^

The variables for analysis were divided into two categories: deformity present and deformity absent. The term ‘true-positive deformity’ referred to a deformity that was initially found by a non-orthopaedic-trained staff member and subsequently confirmed by the paediatric orthopaedic team. The term ‘true-negative deformity’ was defined as the absence of a deformity that was identified by both non-orthopaedic-trained staff and the paediatric orthopaedic team. A deformity that remained undetected by non-orthopaedic-trained staff but was subsequently identified by the paediatric orthopaedic team was classified as a ‘false-negative deformity’. Conversely, a ‘false-positive deformity’ was defined as a deformity detected by non-orthopaedic-trained personnel but undetected by the paediatric orthopaedic team upon subsequent consultation.

The data were analysed descriptively to ascertain whether the cases were representative of the population. Frequencies and percentages were used to present the categorical data, whereas means and standard deviations were utilised to express the continuous data. The threshold for statistical significance was set at P<0.05. Cohen’s kappa coefficients were calculated to assess the reliability of the NCSC, following the criteria set by Landis and Koch^18^ to determine the level of agreement between the non-orthopaedic-trained staff and the paediatric orthopaedic team regarding their findings for each item in the NCSC. Additionally, sensitivity and specificity analyses were conducted to evaluate the validity of the tool.

### Sample size planning

The sample size statement was based on criteria from a previous study.^19^ This study aimed to determine the sensitivity and specificity of the NCSC in identifying newborns with foot deformities. The sample size was calculated using a formula derived from the sensitivity and specificity analyses utilising the Power Analysis and Sample Size Software (2020; NCSS, LLC., Kaysville, Utah, USA, ncss.com/software/ pass). Given a prevalence of 0.050 and a sample sensitivity of 0.970, the sample size needed for a two-sided 95% confidence interval with a width of at most 0.200 was 460. Given a prevalence of 0.050 and a sample specificity of 0.970, the sample size needed for a two-sided 95% confidence interval with a width of at most 0.200 was 25. Since the larger sample size was necessary to ensure that both confidence intervals remained within the desired width, a total sample size of 460 was required. The final sample size was adjusted to 512 to account for a non-response rate of 10.0%.

## Results

### Content validation

The initial content validation led to significant modifications based on the feedback from the first expert panel. The initial focus was on the illustrations and labelling of the CTEV components. The primary changes recommended by the second expert panel included enhancing the clarity of the deformity illustrations by adding visual aids including arrows to indicate the direction of the foot deviation such as the exaggerated arch in cavus and circles to highlight specific areas of the deformity such as forefoot adduction. These revisions were complemented by concise descriptions that further clarified each deformity component. The second round of content validation, conducted by the second expert panel, confirmed that the modified tool was clear, accurate and representative of the intended clinical features, with no further revisions necessary.

### Face validation

The face validation confirmed that the NCSC was well-received by the paediatricians and medical officers involved. The participants unanimously agreed on the appropriateness of the font, layout and pictorial representations. They found the descriptions of each item to be clear and relevant. No further amendments were suggested, indicating that the face validity of the tool was deemed satisfactory for use in routine newborn examinations.

### Pilot study

In the pilot study, 42 feet were inspected. The kappa agreement test revealed an excellent reliability of the tool, with an almost perfect kappa coefficient of 1.00 (P<0.001) observed for all items, except for the adduction deformity component, which yielded a kappa coefficient of 0.71 (P<0.001).

A total of 607 newborns (1214 feet) were examined. The majority were girls (n=334, 55%) (Table 2). In the NCSC group, 306 newborns were screened. In the conventional group, 301 newborns were screened. The Sarawakian Malays represented the largest group, followed by the Indigenous Sarawakians, Chinese and other ethnicities. The mean maternal age at delivery was 29.86 years, with a standard deviation of 5.967, ranging from 15 to 44 years (Table 2).

**Table 2:** Results of the kappa agreement test from the pilot study.

Variable	n
Number of feet examined	
• Feet screened using the NCSC	612
• Feet not screened using the NCSC	602
Sex	
• Female	334 (55%)
○ Screened using the NCSC	156
○ Not screened using the NCSC	178
• Male	273 (45%)
○ Screened using the NCSC	150
○ Not screened using the NCSC	123
Ethnicity	
• Sarawakian Malay	340 (56%)
• Indigenous Sarawakian	192 (31%)
• Chinese	59 (10%)
• Others	18(3%)
Maternal age (year)	
• 15-24	122 (20%)
• 25-34	342 (56%)
• 35-44	143 (24%) Mean: 29.86, standard deviation: 5-967

In the NCSC group, CTEV was detected in two newborns by both the non-orthopaedic-trained staff and paediatric orthopaedic team. One newborn presented with unilateral CTEV, while the other newborn exhibited bilateral CTEV, leading to a total of three feet diagnosed with CTEV. A total of 609 feet were deemed to have no CTEV by both the non-orthopaedic-trained staff and paediatric orthopaedic team.

In the conventional group, 37 feet with CTEV were identified by the house officers. After confirmation from the paediatric orthopaedic team, there were only two cases of unilateral CTEV. Both the non-orthopaedic-trained staff and paediatric orthopaedic team did not detect any deformity in the remaining 564 feet. A newborn initially diagnosed with normal feet was later determined by the paediatric orthopaedic team to have a unilateral CTEV, indicating a false-negative case.

The numbers of true-positive, true-negative, false-positive and false-negative CTEV deformities are detailed in Table 3.

**Table 3:** Summary of the screening results with and without the NCSC.

CTEV screening type	True positive	True negative	False positive	False negative
With the NCSC	3	609	0	0
Without the NCSC	2	564	35	1

The adduction component of the tool demonstrated an almost perfect kappa agreement, while both equinus and final diagnosis exhibited perfect agreement (Table 4). However, the assessment of cavus and varus displayed moderate agreement. The kappa coefficients for all items in the checklist ranged from 0.725 to 1.000, with a significant P-value of <0.001.

**Table 4:** Comparison of the kappa agreement of each deformity component in the NCSC by the non-orthopaedic-trained personnel and paediatric orthopaedic team.

Item	Yes n (%)	Yes n (%)	Kappa coefficient	P-value
Cavus	13 (2.19)	574 (97.81)	0.782	
Adduction	65 (10.94)	520 (89.06)	0.927	
Varus	4 (0.67)	587 (99.33)	0.725	<0.001
Equinus	3 (0.51)	591 (99.49)	1.000	
Final diagnosis	3 (0.51)	591 (99.49)	1.000	

There were 5665 live births in SGH during the study period.^20^ The total number of newborns with true-positive CTEV was 5, resulting in an incidence rate of 0.88 per 1000 live births. Based on this incidence rate, the sensitivity, specificity, predictability and positive likelihood ratio between the feet screened using the NCSC and feet not examined using the NCSC are summarised in Table 5.

**Table 5:** Sensitivity, specificity and predictive values for screening with and without the NCSC.

Item	Screening with the NCSC	Screening without the NCSC
Sensitivity	100.00% (15.81%-100.00%)	66.67% (9.43%-99.16%)
Specificity	100.00% (99.40%-100.00%)	94.16% (91.97%-95.90%)
Positive predictive value	100.00% (15.81%-100.00%)	0.99% (0.42%-2.32%)
Negative predictive value	100.00% (99.40%-100.00%)	99.97% (99.85%-99.99%)

In the NCSC group, the sensitivity and specificity were both 100.00%. Both the positive and negative predictive values were also 100.00%. Conversely, the conventional group showed a lower sensitivity of 66.67%. The specificity was 94.16%, which, while high, was still lower than the specificity observed in the NCSC group. The positive predictive value was substantially lower at 0.99%, reflecting a higher rate of false-positive deformities.

## Discussion

The design of the screening tool developed in this study considered several aspects to optimise its usability. Visual complexity was managed with monochromic schematic images of CTEV, arranged in a vertical alignment. Various approaches exist for organising checklist items and response categories, such as the vertical layout, the block format and the T-format.^21^ The impact of these formats on usability was investigated by Zhang et al.,^21^ who studied eye movements and cognitive load in participants as they searched for target items using the different formats. The findings revealed that the vertical format led to smoother eye movements and more efficient information processing. Participants also perceived the vertical format as more logically organised. In the present study, the vertically aligned column in the NCSC addresses the influence of eye movements on the usability of the tool.

This study was conducted from January to June 2021, coinciding with the Conditional Movement Control Order (CMCO) in Sarawak to mitigate the spread of COVID-19.^22^ The relatively small number of true-positive clubfoot cases observed during this timeframe can be reasonably attributed to the influence of interstate travel restrictions implemented during the CMCO. These restrictions likely led to a shift in birthing practices, with transdistrict travel restrictions prompting patients to opt for delivering their offsprings at the nearest hospitals, thereby affecting the total number of live births at the study site. Before the onset of the COVID-19 pandemic in 2019, the study site recorded a total of 11,571 live births. There was a notable decline of 13.7% in the number of live births in 2021 compared to the pre-pandemic year.^20^ Correspondingly, the study documented a 50% reduction in the number of CTEV cases during the study period in comparison to the corresponding period in 2019.^23^ This observation aligns with the findings by Rangasamy et al.,^24^ who also reported a marked reduction in the number of CTEV cases during the pandemic. Rangasamy et al.^24^ also suggested that the pandemic’s impact on healthcare access, birthing patterns and hospital utilisation likely contributed to the reduced number of cases diagnosed in urban-based hospitals. Nonetheless, comprehensive investigations are warranted to gain a deeper understanding of the pandemic’s broader effects on paediatric orthopaedic conditions.

While prenatal diagnosis of clubfoot is becoming more common with the advancement of ultrasonographic equipment and techniques, postnatal confirmation of the diagnosis is still necessary. In limited-resource institutions and smaller rural healthcare units staffed by midwives, the lack of sophisticated ultrasonographic equipment as well as the absence of radiologists makes postpartum clinical examination the primary means of clubfoot diagnosis. Therefore, having a reliable and effective clubfoot screening tool, such as the NCSC, is crucial.

For each item in the NCSC, the kappa inter-rater reliability test showed that the scores obtained by the staff without specialised orthopaedic training were in agreement with those obtained by the paediatric orthopaedic team. This indicates that individuals without specialised knowledge can effectively utilise the tool. The NCSC was developed with the aim of providing an objective approach to identify clubfoot in newborns postnatally, particularly for those who do not possess specialised orthopaedic training. Unlike existing CTEV classification systems, the NCSC requires minimal to no prior training due to its inclusion of a clear schematic representation of the deformity. The purpose of this tool is to screen for CTEV, not to assess the severity of the condition.

Various classification and scoring systems have been proposed to categorise clubfoot deformities based on different anatomical, clinical and imaging parameters. These systems include the Ponseti and Smoley system^25^; Manes, Costa and Innao classification system^26^; Harold and Walker system^27^; Catterall system^28^; Dimeglio system^29^; and Pirani scoring system.^30^ Nevertheless, none of these classification systems have demonstrated superiority over one another. Most of these systems heavily rely on the expertise of the examiner and necessitate specialised training. The aforementioned classification systems require specific tools, such as a goniometer, to measure the range of motion,^25,27^ and demand a detailed assessment of the anatomical and functional abnormalities of the foot.^26,28^ These systems also necessitate a deep understanding of foot biomechanics, flexibility and the degree of correction achieved.^26,27^ Additionally, they evaluate the reducibility of the foot and assess the severity of stiffness or suppleness of the deformity.^25-28^ The Pirani scoring system has achieved widespread acceptance.^31,32^ This system exhibited moderate to substantial inter-observer reliability between a physiotherapy assistant and a paediatric orthopaedic surgeon. However, 1 week of training at a specialised clubfoot centre was necessary to achieve this reliability.^33^ CTEV classification systems utilising radiography and magnetic resonance imaging (MRI) can be resource-demanding. Furthermore, radiography and MRI may yield potentially inaccurate results when applied to immature non-ossified tarsal _bones._^34,35^

In the group that did not undergo screening using the NCSC, 35 false-positive cases and one false-negative case were observed, resulting in a 5.98% diagnostic error rate. A study conducted in Uttarakhand, India, highlighted the misdiagnosis of clubfoot, revealing that 33.68% of suspected clubfoot cases were non-clubfoot conditions.^8^ Conversely, no misdiagnosis was noted in the NCSC group in the current study.

The sensitivity and specificity of the NCSC were both 100.00%, indicating its high accuracy in correctly identifying true-positive and true-negative CTEV cases. This contrasted with the conventional screening method, which had a sensitivity of 66.67%, failing to detect a third of true-positive cases, and a much lower positive predictive value of 0.99%, indicating many false-positive cases. The high sensitivity of the NCSC ensures that all true-positive CTEV cases are identified, reducing misdiagnoses, while its perfect specificity minimises false-positives and unnecessary referrals. With a positive predictive value and a negative predictive value of 100.00%, the tool ensures that all identified cases are true-positives and that cases not identified do not have CTEV. Therefore, using the NCSC enhances the detection of CTEV in newborns without the need for specialised training or sophisticated imaging facilities. This empowers junior healthcare professionals to identify clubfoot deformities during routine postnatal checks. Implementing the NCSC in resource-limited settings can facilitate early diagnosis, leading to timely interventions.

The present study encountered several limitations that warrant consideration. For instance, the repeated examination by the observers may have led to increasing familiarity with the screening tool over time, potentially resulting in improved performance in subsequent examinations. It may be beneficial to develop multiple versions of the checklist with slight variations in wording or structure, rotating these versions among observers, to mitigate this bias in future studies. This approach could help prevent observers from becoming overly familiar with a single format, thus reducing the likelihood of biased assessments.

Despite successfully achieving the minimum required sample size, the study was challenged by the notably low incidence rate of CTEV during the study period. Additionally, while the NCSC was designed to aid non-orthopaedic-trained healthcare professionals in identifying CTEV, its application was limited to house officers. Therefore, the generalisability of the tool’s effectiveness across other non-orthopaedic-trained healthcare professionals, such as midwives, nurses or primary care physicians, remains unexplored.

Future research could address the study limitations by expanding the sample size, extending the study duration and adopting a multicentred approach. To ensure that familiarity does not significantly affect the results, multiple observers should independently assess each case, allowing for the evaluation of intra-observer reliability. Moreover, expanding the study to include different groups of non-orthopaedic-trained healthcare professionals, such as midwives, community nurses and paramedics, would provide valuable insights into potential variations in observations and enhance the tool’s reliability across various healthcare disciplines and environments.

## Conclusion

This study developed and validated the NCSC to provide a graphical tool for non-orthopaedic-trained practitioners to improve postnatal detection of CTEV. The checklist demonstrates high sensitivity, specificity and positive and negative predictive values, thereby assisting healthcare professionals in the identification of CTEV. The findings suggest that the NCSC offers a more reliable screening approach for detecting postnatal CTEV than conventional methods. Notably, the NCSC is not designed to assess the severity of CTEV but rather to aid in its initial detection.

## Data sharing statement

Data are available upon request to the corresponding author.

**How does this paper make a difference in general practice?**

- This study introduces the Newborn Clubfoot Screening Checklist (NCSC), a tool specifically designed for non-orthopaedic-trained healthcare professionals to detect postnatal congenital talipes equinovarus.
- The high sensitivity and specificity of the NCSC ensure accurate identification of true-positive and true-negative cases, reducing misdiagnoses and unnecessary referrals.
- Implementing the NCSC in general practice can empower junior healthcare professionals, particularly those in resource-constrained settings, enhancing their ability to conduct reliable and efficient postnatal screening.
